# Investigating Spatial Patterns of Pulmonary Tuberculosis and Main Related Factors in Bandar Lampung, Indonesia Using Geographically Weighted Poisson Regression

**DOI:** 10.3390/tropicalmed7090212

**Published:** 2022-08-26

**Authors:** Helina Helmy, Muhammad Totong Kamaluddin, Iskhaq Iskandar, Suheryanto

**Affiliations:** 1 Graduate School of Environmental Science, Sriwijaya University, Palembang 30139, Indonesia; 2 Department of Environmental Sanitation, Tanjung Karang Health Polytechnic, Bandar Lampung 35145, Indonesia; 3 Department of Pharmacology, Faculty of Medicine, Sriwijaya University, Palembang 30114, Indonesia; 4 Department of Physics, Faculty of Mathematics and Natural Sciences, Sriwijaya University, Indralaya 30662, Indonesia; 5 Department of Chemistry, Faculty of Mathematics and Natural Sciences, Sriwijaya University, Indralaya 30662, Indonesia

**Keywords:** infectious disease, epidemiology, health, geographic information system, spatial science, geostatistics

## Abstract

Tuberculosis (TB) is a highly infectious disease, representing one of the major causes of death worldwide. Sustainable Development Goal 3.3 implies a serious decrease in the incidence of TB cases. Hence, this study applied a spatial analysis approach to investigate patterns of pulmonary TB cases and its drivers in Bandar Lampung (Indonesia). Our study examined seven variables: the growth rate of pulmonary TB, population, distance to the city center, industrial area, green open space, built area, and slum area using geographically weighted Poisson regression (GWPR). The GWPR model demonstrated excellent results with an R^2^ and adjusted R^2^ of 0.96 and 0.94, respectively. In this case, the growth rate of pulmonary TB and population were statistically significant variables. Spatial pattern analysis of sub-districts revealed that those of Panjang and Kedaton were driven by high pulmonary TB growth rate and population, whereas that of Sukabumi was driven by the accumulation of high levels of industrial area, built area, and slums. For these reasons, we suggest that local policymakers implement a variety of infectious disease prevention and control strategies based on the spatial variation of pulmonary TB rate and its influencing factors in each sub-district.

## 1. Introduction

Tuberculosis (TB) is a major cause of global health problems, representing one of the leading causes of death due to infectious diseases worldwide (Table A1) [1]. TB is fundamentally caused by Mycobacterium tuberculosis, which affects the lungs (pulmonary TB) while also affecting other sites (extrapulmonary TB) [2]. An acid-fast bacillus (AFB) positive smear is an early-stage indicator while diagnosing pulmonary TB [3]. Moreover, AFB bacterium can cause a host of other infections in addition to TB [4]. Fundamentally, there are several factors, causing pulmonary TB including geographical factors (e.g., altitude) [5,6], environmental factors (e.g., soil moisture) [7], and socio-economic factors (e.g., population density) [5,7,8,9,10].

Auchincloss et al. [11] have reviewed several methods used for epidemiological spatial analysis, such as trends over time, distance calculations, spatial aggregation, clustering, spatial smoothing and interpolation, and spatial regression. Moreover, other studies have focused on the spatial analysis of TB by using the Global Moran’s I and Getis-Ord Gi* autocorrelation statistics to detect the spatiotemporal patterns of TB [9,12,13,14,15]. Meanwhile, Li [16] used the Bayesian spatiotemporal model to analyze the correlation of socio-economic, health, demographic, and meteorological factors with the population level of TB. Other studies have utilized a weight-rating and multi-criteria decision-making score model to map TB risk areas [17].

Several studies have analyzed the spatial interaction between socio-economic factors and pulmonary TB cases by comparing the ordinary least squares (OLS) model and the geographically weighted regression (GWR) model [18,19]. Their results confirmed that the GWR model could better distinguish the relationship between the mean number of smear-positive TB cases and their socio-economic determinants. Hailu et al. [20] have applied Getis-Ord Gi* and the GWR method to explore the spatial cluster patterns of pulmonary TB cases. In this way, they have assessed the spatial heterogeneity with the predictor variables. Despite these advancements, the in-depth analysis of the spatial distribution in urban areas is lacking.

By 2020, 30 countries with the highest TB cases accounted for 86% of TB cases worldwide. Eight of these countries accounted for two-thirds of the global total: India (26%), China (8.5%), Indonesia (8.4%), the Philippines (6.0%), Pakistan (5.8%), Nigeria (4.6%), Bangladesh (3.6%), and South Africa (3.3%) [1]. As per World Health Organization data from 2020 [21], 10 million people worldwide suffer from TB, and 1.2 million people die every year. Global efforts are being made to end the TB epidemic by 2030 (Sustainable Development Goal (SDG) 3.3) by detecting and treating TB cases [22]. The strategies and SDGs imply achieving and targeting large-scale reductions in the incidence of TB, the absolute number of TB deaths, and the costs, faced by TB patients [1].

Indonesia is one of the countries with the highest TB case load globally, with an estimated number of infections reaching 845,000, and a mortality rate of 98,000, equivalent to 11 deaths/h [21]. From a regional perspective, the TB case detection rate for all the TB cases in Lampung Province (Indonesia) has increased by 25–54% from 2017 to 2019 [23]. In particular, the third highest case detection rate was identified in Bandar Lampung with 63% of the detected cases [23], which is the capital city of the Lampung Province and serves as the center of both government, and social, political, economic, educational, and cultural activities [24]. Given the regional importance of Bandar Lampung and abundant TB cases within the city, it is required to elucidate their distribution and drivers toward achieving SDG 3.3. However, the studies about local cases of pulmonary TB and its drivers in Bandar Lampung are lacking.

To fill these research gaps, our study investigates the factors and distribution of pulmonary TB cases in Bandar Lampung using land use and socio-demographic variables. Firstly, we conducted correlation and scatter plot analysis to identify potential variables. Secondly, we used OLS to develop a multivariate equation for pulmonary TB cases estimation using the geographically weighted Poisson regression (GWPR) method. In this case, we assessed GWPR model performance and significance variables based on the statistical report. Thirdly, we analyzed the spatial patterns of pulmonary TB cases and its influencing factors by sub-districts. As a novelty, this study provides high accuracy of the GWPR model and an in-depth spatial patterns analysis of pulmonary TB cases in Bandar Lampung. Furthermore, local and national policymakers can adopt the research findings to control pulmonary TB transmission across the urban area.

## 2. Materials and Methods

### 2.1. Study Area

**Figure 1:**
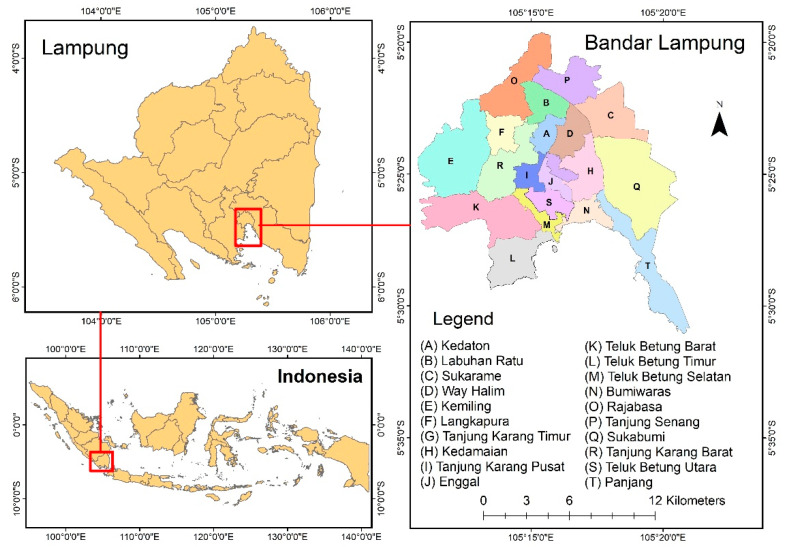
Administration map of Bandar Lampung, Indonesia.

Bandar Lampung (Figure 1) has an area of 197.22 km^2^ with a population density of approximately 6008 people/km^2^ and a population growth rate of 2.16% per year from 2011 to 2021 [25]. Its population growth will reach 1.8 million people by 2030 [26]. As the capital city of the Lampung Province, Bandar Lampung has the highest incidence of TB cases in the province [27]. In 2010, from a pool of 13,533 inhabitants, 1353 were found to be AFB smear-positive [28]. In 2011, the Bandar Lampung had 1314 TB cases, including 1000 smear-positive cases.

### 2.2. Spatial Data Used in This Study

This study used the available spatial data, including land use and socio-demographic data summarized in Table 1.

#### 2.2.1. Socio-Demographic Data

The number of pulmonary TB cases is tuberculosis patients data in 2015 and 2020 was sourced from the Bandar Lampung City Health Office [29]. The pulmonary TB growth rate is the growth of TB cases calculated based on pulmonary TB cases in 2015 and 2020 sourced from the Bandar Lampung City Health Office [29]. The population is a census data of the Bandar Lampung population in 2020, which was carried out every ten years [24].

#### 2.2.2. Land Use Data

Distance to the urban center is data on the distance to the capital by sub-district in Bandar Lampung, which was collected from the government of Bandar Lampung. The distance is based on the unit length of km [24]. The industrial area is data on the area of industrial areas in Bandar Lampung in 2020, which was archived by the Regional Development Planning Agency of Bandar Lampung City [30]. Green open space area are data on the area of green open space in Bandar Lampung, which is sourced from the Regional Development Planning Agency of Bandar Lampung City based on the interpretation of SPOT 6 imagery [30]. The slums area includes data on the area of slums in Bandar Lampung City in 2020, sourced from the Regional Development Planning Agency of Bandar Lampung City [30]. Bult-up areas are obtained to Global Artificial Impervious Areas (GAIA) data products. GAIA uses the complete Landsat archive with a spatial resolution of 30 m on the Google Earth Engine platform. Data are available from 1985 to 2018. Additional data sets include night light data and Sentinel-1 Synthetic Aperture Radar Data. A cross-product comparison shows the GAIA data are the only data set spanning more than 30 years. The temporal trends in GAIA are in good agreement with other datasets at local, regional, and global scales [31].

### 2.3. Methodology

#### 2.3.1. Scatter Plot and Correlation Analysis

The variables influencing pulmonary TB were selected based on scatterplots and correlation analyses. The scatterplot graph and correlation coefficient can be used for identifying required variables based on the strength of the relationship between the two variables. The correlation coefficient is calculated by:

(1) $$r_{xy}=\frac{n\sum xy-(\sum x)(\sum y)}{\sqrt{\left\{n\sum x^{2}-{\left(\sum x\right)}^{2}\right\}\{n\sum y^{2})-{\left(\sum y\right)}^{2}\}}}\;$$

where *r_xy_* is the correlation coefficient, n is the number of data points, ∑x,∑y is the number of each variable, ∑xy is the sum of the multiplication of the variables *x* and *y*. $\sum x^{2},\sum y^{2}$ is the sum of the squares of *x* and *y*.

#### 2.3.2. Ordinary Least Square (OLS)

In this study, OLS analysis was applied to determine spatial dependencies in regression analysis to avoid unstable parameters, to perform significance tests, and to obtain the information about the spatial relationship between the parameters involved in the model [12,32]. Equation (2) formalizes the OLS regression model:

(2) $$Y_{i}=\beta_{0}+\beta_{1}X_{1i}+\beta_{2}X_{2i}+\ldots +\beta_{n}X_{ni}+\varepsilon_{i}\;$$

where $Y_{i}$ is the dependent variable, $X_{1i}$, $X_{2i}$,…$X_{ni}$ are the independent variables, $\varepsilon_{i}$ represents an error, $\beta_{0},$ and $\beta_{1}$…$\beta_{n}$ are the respective intercepts and coefficients [33].

#### 2.3.3. Geographically Weighted Poisson Regression (GWPR)

This study used GWPR to improve the predictions for each sub-district based on the observations in nearby sub-districts. In a GWPR, the pulmonary TB cases are predicted by a set of explanatory variables allowing the parameters to vary over space [34]. The function of the GWPR equation is formalized in Equation (3):

(3) $$\;\ln\left(Y\right)=\ln\left(\beta_{0}\left(u_{i}\right)\right)+\beta_{1}\left(u_{i}\right)X_{1}+\beta_{2}\left(u_{i}\right)X_{2}+\ldots +\beta_{n}\left(u_{i}\right)X_{n}+\varepsilon_{i}$$

where $\beta_{n}\;$ represents the function of location, and $u_{i}=(u_{xi},u_{yi})\;$ denotes the two-dimensional coordinates of the *i*th point in space. This means that the parameter $\beta_{n}=(\beta_{0},\beta_{1},$…$,\beta_{n})$, as estimated in Equation (3), may differ between sub-districts. Thus, in the GWPR method, the parameter $\beta_{n}$ can be expressed by using Equation (4):

(4) $$\beta =\left[\begin{array}{cccc} \beta_{0}(u_{x1},u_{y1}) & \beta_{1}(u_{x1},u_{y1}) & \cdots & \beta_{n}(u_{x1},u_{y1})\\ \beta_{0}(u_{x2},u_{y2}) & \beta_{1}(u_{x2},u_{y2}) & \cdots & \beta_{n}(u_{x2},u_{y2})\\ \cdots & \cdots & \cdots & \cdots \\ \beta_{0}(u_{xk},u_{yk}) & \beta_{1}(u_{xk},u_{yk}) & \cdots & \beta_{n}(u_{xk},u_{yk}) \end{array}\right]$$

where *k* is the number of sub-districts. The parameters for each sub-district, which form a row in Equation (4), are estimated as follows [35]:

(5) $$\hat{\beta }\left(i\right)={\left(X^{T}W\left(u_{xi},u_{yi}\right)X\right)}^{-1}X^{T}W\left(u_{xi},u_{yi}\right)Y$$

In Equation (5), $W\left(u_{xi},u_{yi}\right)$ represents an *n* by *n* spatial weight matrix that can be expressed as W(i)*:*

(6) $$W\left(i\right)=\left[\begin{array}{cccc} w_{i1} & 0 & \cdots & 0\\ 0 & w_{i2} & \cdots & 0\\ \cdots & \cdots & \cdots & \cdots \\ 0 & \cdots & \cdots & w_{ik} \end{array}\right]$$

where $w_{ij}$ (j=1,2,…,k) is the weight given to the sub-district *j* in the model adjustment for sub-district *i*.

#### 2.3.4. Model Assessment

In this study, the evaluation phase of the model accuracy for each sub-district was carried out based on the analysis of the standard deviation of the OLS and GWPR model by using Equation (7):

(7) $$S=\sqrt{\frac{\sum_{1-n}^{n}\;{\left(X_{i}-\bar{X}\right)}^{2}}{n}}$$

where *S* is the standard deviation, *n* is the amount of data, $X_{i}$ is the variance value, and $\bar{X}$ is the calculated average [36]. Moreover, the model fitness was evaluated based on the value of R^2^ and adjusted R^2^, as shown in Equations (8) and (9), respectively:

(8) $$R^{2}=\frac{SSR}{SST}$$

where *SSR* is the square of the difference between the predicted Y value and the average value *Y =* $\overset{n}{\underset{i=1}{\sum }}\;$*(ŷᵢ − ӯ)^2^*, and *SST* is the square of the difference between the actual Y value and the average value *Y =* $\overset{n}{\underset{i=1}{\sum }}\;$*(yᵢ – ӯ)^2^* [37].

(9) $${R^{2}}_{adj}\;=\;1-\frac{MSE}{MST}=1-\left(1-R^{2}\right)\left(\frac{n-1}{n-p-1}\right)\;$$

Here, *MSE* is the mean squared error, *MST* is the total mean squared error, *n* is the number of observations, and *p* is the number of variables [37].

#### 2.3.5. Investigating Spatial Patterns of Incidence Rate and Main Variables

The number of pulmonary TB cases per sub-district, generated by using GWPR, was applied to quantify the incidence rate by Equation (10).

(10) $$Incidence\;rate=\frac{TB\;Case}{Population}\times 100,000\;$$

At the last step, the incidence rate data and main variables were analyzed in depth to determine the spatial distribution pattern and factors, supporting TB cases in each sub-district of Bandar Lampung.

## 3. Results

### 3.1. Correlation and OLS of AFB Smear-Positive Pulmonary TB

**Figure 2:**
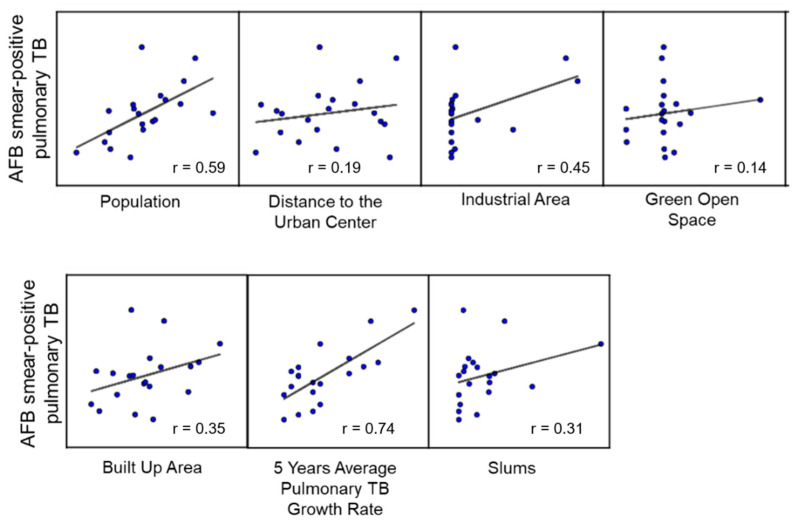
Scatter plot and correlation coefficient of all explanatory variables.

Figure 2 shows the scatter plot and correlation coefficient analysis, reflecting the relationship between each variable and the cases of pulmonary TB. The pulmonary TB growth rate revealed the highest correlation coefficient (0.74) with the number of pulmonary TB cases. On the other hand, several other variables revealed weaker relationships, such as population (0.59), industrial area (0.45), built area (0.35), and slum area (0.31). In contrast, several variables were found to have no correlation with pulmonary TB cases, including distance to the urban center (0.19) and green open space (0.14).

Table 2 summarizes the statistical results of the overall OLS model. In this case, the variance inflation factor (VIF) was further applied to reflect the redundancy between variables, and if the VIF value was more than 7.5, it must be removed. This analysis revealed that the VIF values ranged from 1.352 to 3.678, thereby indicating no redundancy between the explanatory variables used in the study. The coefficient value indicates that several variables positively influenced the rate of pulmonary TB cases, including pulmonary TB growth rate, slum areas, and population. At the same time, several variables showed negative effects on the rate of pulmonary TB cases, including green open space and distance to urban center.

Table 2 also depicts that the growth rate of pulmonary TB cases and population were identified as significant variables for the regression model with the *p*-value of 0.0001 and 0.006, respectively. The model performance indicators revealed that the R^2^ and adjusted R^2^ were 0.83 and 0.73. This indicates that the OLS model had significant properties and was a good fit.

The F-statistic and Joint Wald statistics indicators were conducted to reflect the overall statistical significance of the model. The null hypothesis for both tests is that the model’s explanatory variables are ineffective. The probability value of the Joint F-statistic obtained was 0.001, while the probability value of the Joint Wald statistics was 0.0001. The value of the Joint F-statistic and Joint Wald statistics obtained was <0.05, thereby indicating that the resulted model was statistically significant. The Koenker (BP) statistic was conducted to determine relationship consistency in the model between the explanatory variables and the dependent variable in geographic and data space. The null hypothesis for this test is that the model is stationary. The probability value of the Koenker statistics (BP) obtained in this model was 0.212, thereby suggesting that heteroscedasticity and non-stationarity were not statistically significant. The Jarque–Bera statistic was conducted to determine the statistical distribution of the residuals. The null hypothesis for this test is that the residuals are normally distributed. The probability value of the Jarque–Bera statistics obtained from this model was 0.639. This indicates that the residuals were normally distributed and had no bias. 

Equation (9) illustrates how the seven variables were applied to estimate the spread of pulmonary TB:

(11) $$\begin{array}{cc} Y= & 0.002X_{1}-3.416X_{2}+0.167X_{3}-40.034\;X_{4}-8.995X_{5}+\\ & 5.615X_{6}+0.249X_{7}-7.420 \end{array}$$

The seven variables included population (*X*_1_), distance to the urban center (*X*_2_), industrial area (*X*_3_), green open space (*X*_4_), built area (*X*_5_), five-years average pulmonary TB growth rate (*X*_6_), and slum area (*X*_7_).

### 3.2. Estimation of Pulmonary TB Cases Based on GWPR Method

**Figure 3:**
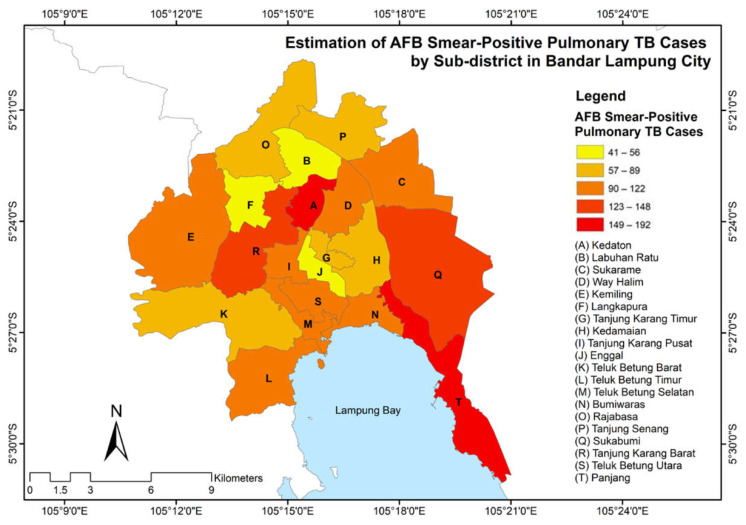
Spatial distribution of AFB smear-positive pulmonary tuberculosis (TB) cases in Bandar Lampung in 2020.

Figure 3 shows that the estimated pulmonary TB cases, obtained from the GWPR processing, were divided into five classes. The very high, high, medium, low, and very low classes accounted for the ranges of 149–192 cases, 123–148 cases, 90–122 cases, 57–89 cases, and 41–56 cases, respectively. Several sub-districts, including Kedaton, Sukabumi, and Panjang, were characterized by relatively higher number of pulmonary TB cases compared to the average cases of all sub-districts. Moreover, a lower number of cases was identified in some sub-districts, such as Labuhan Ratu, Langkapura, and Enggal.

**Figure 4:**
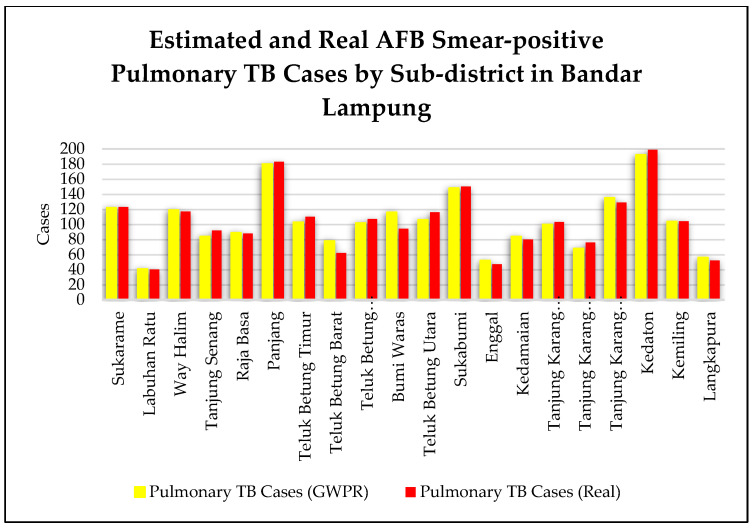
Estimated and real AFB smear-positive pulmonary tuberculosis (TB) by sub-districts in Bandar Lampung.

The comparison of the estimated and real number of AFB smear-positive pulmonary TB cases for each sub-district in Bandar Lampung is shown in Figure 4. The average error of AFB smear-positive pulmonary TB cases in all the Bandar Lampung sub-districts was six cases. The Bumi Waras and Teluk Betung Barat sub-districts were characterized by high error, above 15 cases, while the lowest error was identified in the Kemiling sub-district, with 0 cases.

### 3.3. Statistical Analysis of GWPR Model

The number of neighbors obtained from the GWPR model indicates that the optimal number of adaptive neighbors in this model was 15. The sigma-squared obtained in this model was 72,329.187, thus indicating that this model matched the observed data well. The value of deviance explained by the local vs. global model was 0.716, thereby suggesting that the local model performed better than the global model. The AICc value was found to be lower than OLS (195.456). In general, the statistical indicators of GWPR clearly indicate that the estimated GWPR model had significant properties with the R^2^ and adjusted R^2^ of 0.96 and 0.94.

**Figure 5:**
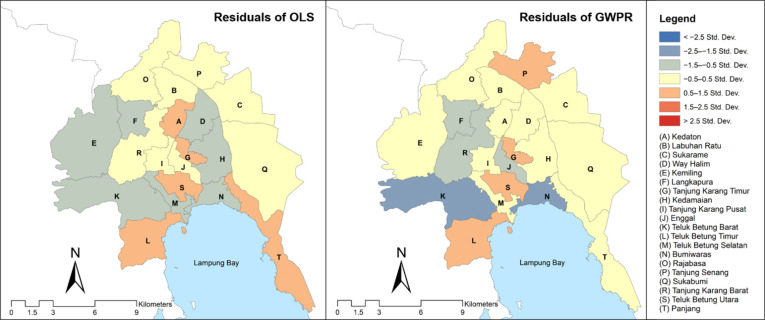
Residuals map of OLS and GWPR model.

Figure 5 demonstrates that visually, the residuals of GWPR were lower than the residuals of OLS. The residuals of the GWPR model were in the range of −1.5 to 1.5 Std. Dev. We identified only two sub-districts in the range of <−2.5 Std. Dev. (the Teluk Betung Barat and Bumi Waras sub-districts). Teluk Betung Barat had a reasonably medium overprediction value due to the high number of pulmonary TB cases affected by the Tanjung Karang Barat and Teluk Betung Selatan. Bumi Waras had also a reasonably medium overprediction value due to the high number of pulmonary TB cases affected by Panjang and Sukabumi. An extremely high or low number of cases in a sub-district could have triggered an overprediction or underprediction in its neighboring sub-districts. However, we discerned somewhat low residuals values in other sub-districts as the GWPR yielded relatively higher R^2^, adjusted R^2^, and AICc than OLS. The relatively small residuals in most sub-districts indicate that the overall number of cases estimated by the GWPR model was close to the actual value.

### 3.4. Spatial Pattern of Pulmonary TB Cases

**Figure 6:**
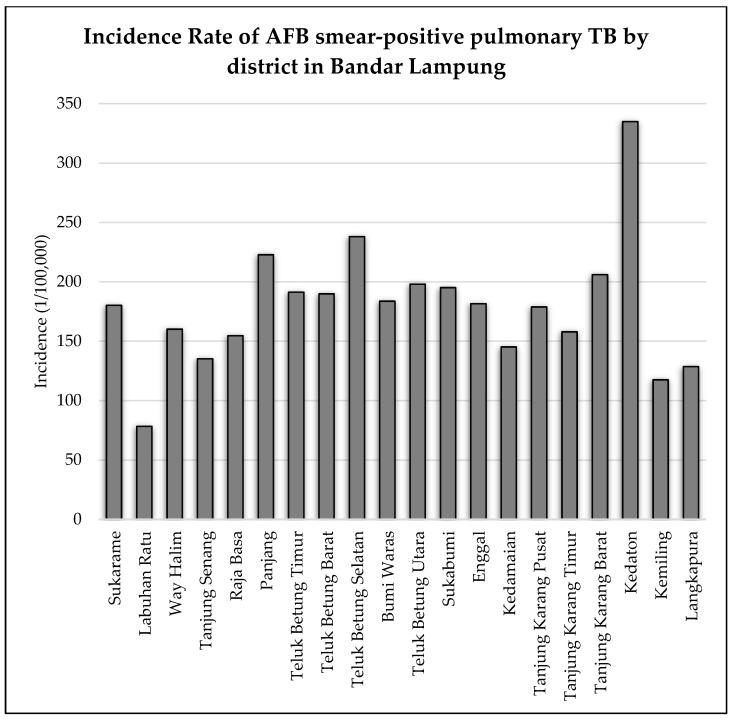
Incidence rate of pulmonary TB in Bandar Lampung in 2020.

As shown in Figure 6, the highest incidence rate of AFB smear-positive pulmonary TB was observed in the Kedaton sub-district, and the lowest incidence rate was observed in the Labuan Ratu sub-district. Notably, several sub-districts with more than 200 incidence rate (Kedaton, Teluk Betung Selatan, Panjang, and Tanjung Karang Barat) require peculiar attention due to the number of pulmonary TB cases.

**Figure 7:**
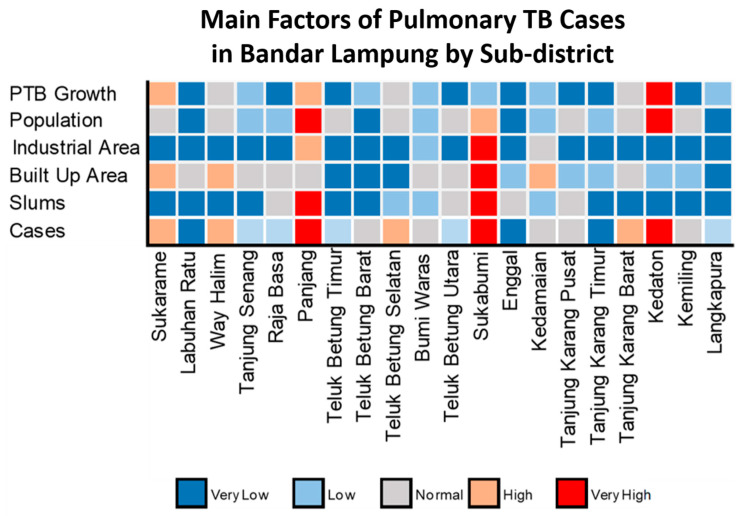
Spatial variations of pulmonary TB cases, pulmonary TB growth rate, population, built area, industrial area, and slums.

Furthermore, we conducted an in-depth analysis of the spatial pattern and elucidated the relationship between the number of pulmonary TB cases and several main variables in each sub-district of Bandar Lampung (see Figure 7).

We identified numerous cases and main variables in the Kedaton, Panjang, and Sukabumi sub-districts. Each sub-district exhibited distinctly characteristics. The number of pulmonary TB cases in Kedaton was strongly affected by the high growth rate of pulmonary TB cases and population. Moreover, Panjang was affected by a high pulmonary TB cases growth rate, as well as high total population, industrial areas, and slum areas. Furthermore, in Sukabumi, pulmonary TB cases were more affected by the population, industrial areas, built areas, and slum areas.

In general, there were no sub-districts with a high rate of pulmonary TB cases, where low levels of main variables were identified. This finding indicates that the factors of pulmonary TB cases in Bandar Lampung were dominated by the pulmonary TB case growth rate, total population, industrial areas, built areas, and slum areas. This pattern is corroborated by the low rate of pulmonary TB cases in several sub-districts with low levels of main variables. Moreover, two sub-districts with a low rate of pulmonary TB cases and low main variable were also identified (Langkapura and Tanjung Karang Timur).

## 4. Discussion

In general, pulmonary TB growth rate and population were the two dominant factors of pulmonary TB cases. The pulmonary growth rate of TB has a strong correlation, while the population has a moderate correlation. OLS statistics also confirmed that these variables were statistically significant with the *p*-value < 0.01. Spatial pattern analysis revealed that high pulmonary TB cases in the Kedaton and Panjang were driven by the high pulmonary TB growth rate and population. According to some previous research, the growth rate of pulmonary TB cases and population has a large influence on the number of pulmonary TB disease [18,38,39,40,41]. Increasing urban population density and scarce health resources may contribute to the gradual expansion of the pulmonary TB epidemic. This may be so because economic development greatly promoted public transportation, which provided convenience for population mobility and opportunities for spatial transmission of pathogens [19,42].

The results showed that industrial areas, built areas, and slum areas had a weak correlation and insignificant with pulmonary TB cases. However, based on the findings of similar studies, a more reasonable explanation is that slum settlement is one of the variables influencing the distribution of pulmonary TB diseases [43,44,45]. This is reasonable because slum environments create a conducive environment for TB spread due to high population density and lack of basic amenities such as decent housing, access to clean water, lack of drainage, and basic sanitation. Furthermore, some of the TB-related conditions which are likely to occur in slums areas include ineffective health services in crowded and poorer populations, poor patient compliance, a large pool of untreated cases, delayed diagnosis, and inappropriate treatment regimens [46,47]. From spatial pattern analysis, it was also identified that in Sukabumi, the number of pulmonary TB cases was high due to the high built, industrial, and slums area. In this case, the different categorization of regions in Sukabumi may have caused a difference in the findings. As an illustration, industrial and slum areas in Bandar Lampung are only identified in particular sub-districts, i.e., Sukabumi and Panjang.

Overall, it can be concluded that sub-districts with a high rate of pulmonary TB cases tend to have a high pulmonary TB growth rate and population. However, several sub-districts with high-rate pulmonary TB cases identified a high level of the industrial, built, and slums area. For these reasons, it is clear that the dominant factors of pulmonary TB cases may vary geographically and can be an accumulation of several factors. This finding indicates that the local government should put extra effort into sub-districts that are densely populated and have a high growing rate of pulmonary TB cases. Rather than just pulmonary TB growth and population, other main variables also affect pulmonary TB cases. Hence, this should lead to various strategic approaches to controlling pulmonary TB transmission [48,49,50].

The GWPR model shows excellent results with an R^2^ and adjusted R^2^ of 0.96 and 0.94, respectively. Based on previous research, the model demonstrated more accurate results according to the higher R^2^ produced in several previous studies [18,20,40,51]. Our high values of R^2^ and adjusted R^2^ imply that the developed model can better represent the spatial variation of pulmonary TB cases in Bandar Lampung. This can be used to analyze pulmonary TB cases control strategies by simulating the number and variables. Moreover, variables applied in this study can also be utilized as a basis for developing further pulmonary TB case spatial models in other urban areas.

However, there was a noticeably high difference between R^2^ and adjusted R^2^ of the OLS model (the adjusted R^2^ is 0.1 point lower). The difference of 0.1 point was identified due to several less relevant variables, causing the adjusted R^2^ to decrease. To alleviate these statistical shortcoming, future studies should thoroughly consider several variables that significantly affect increasing pulmonary TB cases at the city scale. To this end, Sun et al. [40] stated that environmental factors, climatic factors, and rainy days have a complex impact on increasing the prevalence of TB. Other studies have revealed that temperature, humidity, and sunlight also affect Mycobacterium tuberculosis growth [52,53,54]. Previous studies also suggested that pollution may increase the risk of pulmonary TB in the urban center of the industrial area [55,56]. Therefore, environmental, climatic, and air quality indicators can be explored to analyze their relation to pulmonary TB cases [57]. In this case, in situ data measurement can be collected in some areas to study its relation to pulmonary TB cases on a local scale [52,53,54]. Some research articles also report the number of other infectious disease cases, income per capita [18,40,51], the number of industrial workers, sanitation quality, HIV prevalence, child mortality, smoking, and diabetes rates, which are additional factors associated with the progression of pulmonary TB [39,58,59,60,61,62]. Therefore, future studies can explore various potential variables to understand the spatial pattern of pulmonary TB cases in urban areas, especially in high incidence rate cities.

In the case of spatial epidemiology, future studies can explore spatial clustering methods, e.g., spatial autocorrelation [63], global Moran’s I statistics, Kulldorff’s scan statistic [64,65], Getis-Ord Gi* [66,67], the generalized linear regression model, and the generalized additive model [68] to analyze the spatial distribution of pulmonary TB. In addition, combining several geospatial techniques with epidemiologically related cases can provide further insight [69]. Furthermore, a spatial risk model of pulmonary TB can be developed based on a disaster mitigation approach by involving hazard, vulnerability, and capacity aspects to assist policymakers in designing intervention targets [20].

## 5. Conclusions

Pulmonary TB is a widespread infectious disease affecting millions of people worldwide every year. Due to the alarming rate of the spread of pulmonary TB, particularly in developing countries, medical professionals are implementing new strategies for reducing the incidence rate and the absolute number of TB deaths. Therefore, this study employed a spatial approach to understand pulmonary TB transmission in Bandar Lampung, Indonesia. Correlation analysis depicted that the growth rate of pulmonary TB and population have strong and moderate positive correlations, respectively, with the number of pulmonary TB cases. Analysis by OLS also confirmed that these variables are statistically significant with the *p*-value < 0.01. Moreover, the GWPR model demonstrated a reliable result with an R^2^ and adjusted R^2^ of 0.96 and 0.94, respectively. The GWPR model developed in this study can help to simulate the current status and future direction of pulmonary TB transmission. Through spatial analysis, we discovered that the factors of high pulmonary TB growth rate, large population, and large amounts of built, industrial, and slums areas affect the high-rate pulmonary TB cases in the Kedaton, Panjang, and Sukabumi sub-districts of Bandar Lampung. However, the drivers of each sub-district are spatially varied. The variation in pulmonary TB rate and its influencing factors can lead to different control strategies for each sub-district at the local level. In this case, policymakers should realize that geospatial insight is a critical aspect that needs to be adopted as a part of evidence-based policymaking in epidemiology and outbreak management to achieve community health resilience.

## Figures and Tables

**Table 1:** Characteristics of the spatial data used in this study.

No.	Data	Data Class	Timespan	Reference
1	Number of Pulmonary Tuberculosis Cases	Socio-demographic	2020	[29]
2	Pulmonary Tuberculosis Growth Rate	Socio-demographic	2015–2020	[29]
3	Population	Socio-demographic	2020	[24]
4	Distance to the Urban Center	Land Use	2020	[24]
5	Industrial Area	Land Use	2020	[30]
6	Green Open Space Area	Land Use	2020	[30]
7	Slums Area	Land Use	2020	[30]
8	Built Area (GAIA)	Land Use	1985–2018	[31]

**Table 2:** Statistical summary of ordinary least square (OLS) results.

Variable	Coefficient	StdError	t-Statistics	Probability	Robust_SE	Robust_t	Robust_Pr	VIF
Intercept	−7.420	25.487	−0.291	0.078	19.944	−0.372	0.716	-
Population	0.002	0.001	3.320	0.006 *	0.001	5.773	0.000 *	2.631
Distance to the Urban Center	−3.416	1.975	−1.730	0.109	1.336	−2.556	0.025 *	1.828
Industrial Area	0.167	3.763	0.044	0.965	2.568	0.065	0.949	3.678
Green Open Space	−40.034	56.106	−0.714	0.489	37.453	−1.069	0.306	1.931
Built Area	−8.995	6.864	−1.311	0.215	5.318	−1.691	0.117	2.591
5 Years Average Pulmonary TB Growth Rate	5.615	1.157	4.581	0.000 *	1.195	4.697	0.001 *	1.352
Slums	0.249	0.143	1.735	0.108	0.078	3.190	0.008 *	2.633
**Diagnostics of OLS**								
Number of Observations	20		Akaike’s Information Criterion (AICc)					205.284
Multiple R-Squared	0.83		Adjusted R-Squared					0.73
Joint F-Statistics	8.288		Prob (>F), (7,12) degrees of freedom					0.001 *
Joint Wald Statistics	177.349		Prob (>chi-squared), (7) degrees of freedom					0.000 *
Koenker (BP) Statistics	9.603		Prob (>chi-squared), (7) degrees of freedom					0.212 *
Jarque–Bera Statistics	0.896		Prob (>chi-squared), (2) degrees of freedom					0.639 *

* An asterisk next to a number indicates a statistically significant *p*-value (*p* < 0.01).

## Data Availability

The datasets generated during and/or analyzed during the current study are available from the corresponding author on reasonable request.

## Appendix A

**Table A1:** Estimated number of pulmonary TB deaths in 2020 [1,70,71].

Region	Pulmonary TB Deaths
World	1,500,000
Indonesia	13,174
Lampung	163
Bandar Lampung	32
